# Risk factors for high cerebral blood flow velocity and death in Kenyan children with Sickle Cell Anaemia: role of haemoglobin oxygen saturation and febrile illness

**DOI:** 10.1111/j.1365-2141.2009.07660.x

**Published:** 2009-05

**Authors:** Julie Makani, Fenella J Kirkham, Albert Komba, Tolulope Ajala-Agbo, Godfrey Otieno, Gregory Fegan, Thomas N Williams, Kevin Marsh, Charles R Newton

**Affiliations:** 1 Department of Haematology and Blood Transfusion, Muhimbili University of Health and Allied SciencesDar-es-Salaam, Tanzania; 2 Kenya Medical Research Institute (KEMRI), Centre for Geographic Medicine Research – CoastKilifi, Kenya; 3 Centre for Clinical Vaccinology and Tropical Medicine, Churchill Hospital, University of OxfordSouthampton, London; 4 University of SouthamptonSouthampton, London; 5 Neurosciences Unit, Institute of Child Health, University College LondonLondon; 6 Infectious Disease Epidemiology Unit, Department of Epidemiology and Population; Health, London School of Hygiene & Tropical MedicineLondon; 7 Clinical Research Unit, London School of Hygiene and Tropical MedicineLondon, UK

**Keywords:** Sickle Cell Anaemia, Africa, transcranial Doppler ultrasound, mortality, cerebrovascular accidents

## Abstract

High cerebral blood flow velocity (CBFv) and low haemoglobin oxygen saturation (SpO_2_) predict neurological complications in sickle cell anaemia (SCA) but any association is unclear. In a cross-sectional study of 105 Kenyan children, mean CBFv was 120 ± 34·9 cm/s; 3 had conditional CBFv (170–199 cm/s) but none had abnormal CBFv (>200 cm/s). After adjustment for age and haematocrit, CBFv ≥150 cm/s was predicted by SpO_2_ ≤ 95% and history of fever. Four years later, 10 children were lost to follow-up, none had suffered neurological events and 11/95 (12%) had died, predicted by history of fever but not low SpO_2_. Natural history of SCA in Africa may be different from North America and Europe.

An estimated 230 000 children are born in Africa each year with Sickle Cell Anaemia (SCA) with high reported mortality due to malaria, bacterial infections and anaemia (Fleming, 1989) but no African country in has an established national management policy. Neurological complications are a significant cause of morbidity and mortality. Reports suggest that the incidence of stroke in SCA patients in Africa is as high as 1·3/100 patient years (Izuora *et al*, 1989; Amayo *et al*, 1992) compared with 0·61/100 patient years in the USA (Ohene-Frempong *et al*, 1998), possibly due to higher prevalences of the key risk factors (low haemoglobin, high white cell count, Bantu haplotype) (Ohene-Frempong *et al*, 1998). Abnormal and conditional cerebral blood flow velocity (CBFv) >200 cm/s and 170–199 cm/s respectively, measured by transcranial Doppler (TCD) ultrasonography of the intracerebral arteries, respectively, predicted 40% and 7% risk of stroke over the subsequent three years (Adams *et al*, 1992; Kirkham *et al*, 2001) while CBFv > 150 cm/s may indicate intracranial vessel stenosis (Adams *et al*, 1990). Peripheral haemoglobin oxygen saturation (SpO_2_) is also a predictor of overt stroke (Kirkham *et al*, 2001; Quinn & Sargent, 2008) and might also predict death, since it is associated with pulmonary hypertension (Minniti *et al*, 2009). There has been no previous description of CBFv or SpO_2_ in African SCA patients, their predictors or their use for identifying those at high risk of adverse outcomes. In this report we describe the spectrum of CBFv in a Kenyan paediatric SCA cohort, exploring factors associated with high CBFv and relating these data to mortality.

## Study design

The study started in 2002 in Kilifi District Hospital (KDH), following approval by the Kenya National Ethical committee. There is a dedicated outpatient clinic for all SCA patients, seen every 3–4 months after clinical diagnosis as there is no newborn screening. Proguanil is given as antimalarial prophylaxis but none of the patients are treated with penicillin prophylaxis or Hydroxycarbamide. Study patients were recruited when well. Following informed consent, history of clinical events, such as febrile illness and episodes of neurological compromise including seizures, was obtained, systematic general and neurological examination conducted and daytime SpO_2_ (pulse oximetry; Nellcor, Pleasanton, CA, USA) measured. Laboratory investigations included blood count (MDII; Beckman Coulter, Fullerton, CA, USA) and sickle genotype confirmed by alkaline haemoglobin electrophoresis (Helena, Sunderland, Tyne & Wear, UK). All information was collected in standardized clinical research forms. Patients were followed up to November 2006 through clinic visits, with admissions, neurological events and deaths captured by active surveillance.

CBFv was measured following the STOP (Stroke prevention in sickle cell disease) protocol (Nichols *et al*, 2001) using the Companion II (Nicolet, Warwick, UK) machine. The highest CBFv (time-averaged maximal mean velocity in the distal internal carotid artery and middle cerebral artery on either side) was determined. CBFv was classified as low (<50 cm/s), normal (50–149 cm/s) or high (≥150 cm/s) based on previous criteria (Adams *et al*, 1990) and our Kenyan control data (Newton *et al*, 1996).

STATA 9 (StataCorp, College Station, TX, USA) was used for statistical analysis. Continuous variables were analysed using two-sided *t*-tests. Categorical variables were analysed by chi-squared test. Logistic regression was used to explore associations between CBFv and clinical and laboratory variables, presenting the results as odds ratio (OR) with 95% confidence interval (CI)_._*P*-values <0·05 were considered statistically significant. Multivariate analysis included all variables with univariate significance of <0·1. Using backward elimination, the final model included all variables significant at the 0·05 level. Binary logistic regression was also used to assess factors predicting death (as date of death was not known).

## Results

In 2002, 105 children with SCA (mean ± SD 7·4 ± 4·0 years) had TCD measurements, with a mean CBFv of 120 ± 34·9 cm/s. Seven (7%) SCA patients had low, 80 (76%) normal and 18 (17%) high CBFv. Only 3 (3%) patients had conditional CBFv (170–199 cm/s) and none had abnormally high CBFv (>200 cm/s). The age group with the greatest prevalence of high CBFv was 5–9 years. Thirteen patients (12·4%) had a history of non-febrile convulsions (2 high CBFv, 1 low CBFv), while only one patient (normal CBFv) had a history of stroke. Children with previous neurological complications did not have lower haematocrit (*P* = 0·94) or SpO_2_ (*P* = 0·45) or higher CBFv (*P* = 0·87).

Patients with high CBFv were significantly more anaemic (haematocrit 22·9 ± 4·8% vs. 25·5 ± 4·3%, *P* = 0·04) with lower mean SpO_2_ (97 ± 2·97% vs. 98·5 ± 2·39%, *P* = 0·04) compared to the normal CBFv group (Table I). There was a trend for ≥3 reported febrile episodes in the past year to be associated with high CBFv (*P* = 0·08; Table I). Multivariate analysis using a model that included age and haematocrit showed that SpO_2_ ≤ 95% (*P* = 0·02) and history of fever were associated with high CBFv, with the likelihood of having high CBFv increasing with the number of episodes of reported fever (OR 13·8; *P* = 0·01 for ≥3 reported febrile episodes; Table I).

**Table I:** Factors associated with high CBFv (≥150 cm/s) in SCA patients in Kenya.

	Normal or low CBFv (*n* = 87)	High CBFv (*n* = 18)	Odds ratio (95%CI)	*P*
Mean Age, years (SD)	7·6 (4·3)	6·5 (2·7)	0·93 **(**0·82–1·06**)**	0·31
History of fever in past year, *n* (%)	40 (46)	8 (44)		
1 episode of fever	34 (39)	4 (22)	0·55 (0·16–1·91)	0·35
2 episodes of fever	3 (3)	1 (6)	1·57 (0·15–16·66)	0·71
3 or more episodes of fever	3 (3)	3 (17)	4·7 (0·83–26·77)	0·08
History of chest events, *n* (%)	19 (22)	3 (17)	0·83 **(**0·38–1·79**)**	0·63
Mean Peripheral SpO_2_, % (SD)	98·5 (2·39)	97 (2·97)	0·83 **(**0·69–1·01**)**	0·04
Peripheral SpO_2_ ≤ 95%, *n* (%)	6 (8)	4 (31)	5·48 (1·29–23·18)	0·02
Mean Haematocrit, % (SD)	25·5 (4·33)	22·9 (4·79)	0·85 **(**0·73–0·99**)**	0·04
Multivariate analysis				
Age, years			0·97 (0·81–1·15)	0·71
1 episode of fever in past year			1·1 (0·24–5·03)	0·90
2 episodes of fever in past year			1·87 (0·12–29·1)	0·66
3 or more episodes of fever in past year			13·8 (1·72–111·2)	0·01
Haematocrit, %			0·92 (0·77–1·09)	0·34
Peripheral SpO_2_ ≤ 95%			6·13 (1·27–29·5)	0·02

[Mean (SD)] Geometric mean, standard deviation in parenthesis.

Prevalence of patients in the respective groups.

Odds ratio [95% Confidence Intervals (95% CI)] and *P*-value to show the association between the respective factor and high CBFv.

By 2006, 10 were lost to follow-up, none of whom had a high baseline CBFv or SpO_2_ ≤ 95%. For the 95 children with available data (Table II) none had suffered neurological complications but eleven (12%) had died. There was no significant difference in mortality between those with normal or low CBFv, compared to those with high CBFv (20 vs. 9%*; P* = 0·38). In univariate analysis, history of febrile illness (*P* = 0·02) at baseline and blood transfusion (*P* = 0·04) during follow-up were associated with increased risk of death. Although not statistically significant, survivors had a higher haemoglobin and CBFv, but a paradoxically lower SpO_2_, at baseline. History of febrile illness and blood transfusion were not predictors of death in a multivariate model that also contained peripheral SpO_2_ (Table II).

**Table II:** Factors associated with death in SCA patients in Kenya.

	Patients who Survived (*n* = 84)	Patients who died (*n* = 11)	Odds Ratio (95%CI)	*P*
Mean Age, years (SD)	7·35 (4·1)	6·8 (4·4)	0·97 (0·83–1·13)	0·69
History of fever (Yes/No), *n* (%)	34 (41)	9 (82)	6·62 (1·34–32·54)	0·02
History of blood transfusion, *n* (%)	10 (12)	4 (36)	4·23 (1·04–17·1)	0·04
Mean Peripheral SpO_2_, % (SD)	98 (2·7)	99·6 (0·8)	2·07 (0·94–4·57)	0·07
Mean Haematocrit, % (SD)	24·9 (4·29)	23·6 (3·5)	0·92 (0·77–1·09)	0·34
High CBFv (≥150 cm/s), *n* (%)	17 (20)	1 (9)	0·39 (0·05–3·29)	0·38
Mean CBFv, cm/s (SD)	118·8 (34·4)	117·8 (23·1)	0·99 (0·98–1·02)	0·93
Multivariable analysis*				
History of fever (Yes/No)			3·43 (0·65–18·07)	0·15
History of blood transfusion			2·80 (0·62–12·66)	0·18
Peripheral SpO_2_† (%)			1·90 (0·86–4·20)	0·11

[Mean (SD)] Geometric mean, standard deviation in parenthesis.

Prevalence of patients in the respective groups.

Odds ratio [95% Confidence Intervals (95% CI)] and *P*-value to show the association between the respective factor and death.

* Fourteen children were excluded from the multivariate analysis of death due to missing data.

† Peripheral oxygen saturation was used as a continuous variable as none of the children who died had SpO2 <95%.

## Discussion

This is the first description of CBFv in African SCA children. The lower mean value for CBFv (120 vs. 129 cm/s) and absence of abnormal CBFv, in contrast to the 3–10% prevalence in the USA (Adams *et al*, 1992), are surprising, given the reportedly higher incidence of stroke in Africa. This may be due to rapid progression of disease, with death swiftly following the development of high CBFv in young children, although we found no evidence for any link between CBFv and mortality. In addition to the possibility of an alternative mechanism, such as venous sinus thrombosis, neurological complications in African children with SCA may be secondary to progressively worsening stenosis, with decrease in CBFv and sometimes moyamoya collaterals (Hogan *et al*, 2005). In our study, low CBFv compatible with, but not diagnostic of, moyamoya (Takase *et al*, 1997) was documented in seven SCA patients, one of whom had had convulsions; none of these patients was known to have died or have neurological complications 4 years later.

In univariate analysis, patients with high CBFv had lower haematocrit and oxygen saturation and were more likely to have had ≥3 febrile episodes; however on multivariate analysis, only the latter two factors were independently associated with high CBFv. Recent evidence suggests that low oxygen saturation predicts neurological events (Kirkham *et al*, 2001; Quinn & Sargent, 2008); this might in part be explained by an association between low SpO_2_ and high CBFv but any link to stroke risk cannot be ascertained from our data in view of the low prevalence of conditional or abnormal CBFv and the lack of neurological events.

Although not statistically significant, those who died had a higher SpO_2_ at baseline. In studies in the USA, low SpO_2_ is associated with pulmonary hypertension, a predictor of death in adults, but these relatively young Kenyan children may not have yet developed this chronic complication (Minniti *et al*, 2009). Variation in exposure and adaptation to anaemic and hypoxic hypoxia may explain some of the phenotypic variability in SCA (Kirkham & Datta, 2006).

We report an association between parental report of ≥3 febrile illnesses in the past year and high CBFv, independent of age, haematocrit and SpO_2._ Mortality was also predicted by previous febrile illness. However, cause of death was not recorded so we cannot infer any direct link with infection or exclude neurological causes. The majority of SCD patients do not currently receive penicillin prophylaxis in most African countries, as there are conflicting data regarding the importance of infection, particularly streptococcal septicaemia, as a cause of morbidity and mortality (Makani *et al*, 2007). Assuming that febrile illness is commonly associated with infection, it is important to determine the causal agent(s) so that preventative strategies can be evaluated in this setting.

Given that this was a cross-sectional study conducted in 2002, with historical data about CNS events and febrile episodes and limited information available from clinical review and surveillance up to 2006, it is difficult to accurately determine the cause-effect relationship of the various factors and their change over time. Larger longitudinal population-based birth cohorts, with repeated laboratory measurements, such as CBFv and SpO_2_, and details of acute illness and clinical events before death, are required to identify risk factors and document the natural history of SCA in Africa.

This study shows a novel association between low SpO_2_ and increased CBFv, which should be explored in non-African settings. The absence of documented abnormally high CBFv suggests that the course and mechanism of cerebral vasculopathy and neurological events in Africa are different from those in North America and Europe. More detailed neuroimaging studies with magnetic resonance angiography are required to elucidate this further.
